# Supplementary material for: A meta-analysis contrasting active versus passive restoration practices in dryland agricultural ecosystems
Source: PeerJ. 2020 Nov 23;8:e10428. doi: 10.7717/peerj.10428 (PMC7690292; doi:10.7717/peerj.10428)
Supplement: Supplemental Information 2 [file peerj-08-10428-s002.docx]

Supporting Information

A meta-analysis contrasting active versus passive restoration practices in dryland agricultural ecosystems

M. Florencia Miguel^*^, H. Scott Butterfield, Christopher J. Lortie

^*^Corresponding author

E-mail: fmiguel@mendoza-conicet.gob.ar

**Table S1.** List of published articles included in a meta-analysis comparing active versus passive restoration practices in dryland agricultural – here defined as farmlands and grazing lands – ecosystems globally. Articles were found by systematically searching in Scopus and The Web of Science in September 2018 and then again in January 2020.

| Authors | Title | Year | Journal | Vol. | pp |
| --- | --- | --- | --- | --- | --- |
| Y.L. Zhang *et al*. | Response of soil enzyme activity to long-term restoration of desertified land | 2015 | Catena | 133 | 64-70 |
| Z. Zeng *et al*. | Effects of habitat alteration on lizard community and food web structure in a desert steppe ecosystem | 2014 | Biol. Conserv. | 174 | 86-92 |
| R. Liu *et al*. | Effect of naturally vs manually managed restoration on ground-dwelling arthropod communities in a desertified region | 2014 | Ecol. Engineer. | 73 | 545-552 |
| E. Huber-Sannwald and D.A. Pyke | Establishing Native Grasses in a Big Sagebrush–Dominated Site: An Intermediate Restoration Step | 2005 | Restor. Ecol. | 13 | 292-301 |
| R.T. Huddleston and T.P. Young | Spacing and Competition Between Planted Grass Plugs and Preexisting Perennial Grasses in a Restoration Site in Oregon | 2004 | Restor. Ecol. | 12 | 546-551 |
| G. Browna and S. Al-Mazrooei | Rapid vegetation regeneration in a seriously degraded *Rhanterium epapposum* community in northern Kuwait after 4 years of protection | 2003 | J. Environ. Manag. | 68 | 387-395 |
| Z. Shang *et al*. | The response of soil organic carbon and nitrogen 10 years after returning cultivated alpine steppe to grassland by abandonment or reseeding | 2014 | Catena | 119 | 28-35 |
| F. Caravaca *et al*. | Establishment of shrub species in a degraded semiarid site after inoculation with native or allochthonous arbuscular mycorrhizal fungi | 2003 | App. Soil Ecol. | 22 | 103-111 |
| L.L. Jackson *et al*. | Desert Restoration | 1991 | Restor. Manag. Notes | 9 | 71-79 |
| Z. Yuan *et al*. | Effects of legume species introduction on vegetation and soil nutrient development on abandoned croplands in a semi-arid environment on the Loess Plateau, China | 2016 | Sc. Total Environ. | 541 | 692-700 |
| M.J. Banerjee | Native Plant Regeneration on Abandoned Desert Farmland: Effects of Irrigation, Soil Preparation, and Amendments on Seedling Establishment | 2006 | Restor. Ecol. | 14 | 339-348 |
| C.M.C.S. Listopad | The effect of grazing exclusion over time on structure, biodiversity, and regeneration of high nature value farmland ecosystems in Europe | 2018 | Sc.Total Environ. | 610 | 926-936 |
| A. Koyama *et al*. | Response of degraded vegetation to introduction of prescribed burning or mowing management in a Mongolian steppe | 2015 | Grassland Science | 62 | 37-44 |
| Z. Yuan *et al*. | Factors affecting the recovery of abandoned semi-arid fields after legume introduction on the Loess Plateau | 2015 | Ecol. Engineer. | 79 | 86-93 |
| A. Yanagawa *et al*. | Factors limiting vegetation recovery processes after cessation of cropping in a semiarid grassland in Mongolia | 2016 | J. Arid Environ. | 131 | 1-5 |
| S.D. Wilson | Managing contingency in semiarid grassland restoration through repeated planting | 2015 | Restor. Ecol | 23 | 385-392 |
| S.M. Munson and W.K. Lauenroth | Plant Community Recovery Following Restoration in Semiarid Grasslands | 2011 | Restor. Ecol. | 20 | 656-663 |
| D. Kinyua *et al*. | Short-Term and Long-Term Effects of Soil Ripping, Seeding, and Fertilization on the Restoration of a Tropical Rangeland | 2009 | Restor. Ecol. | 18 | 226-233 |
| Z. Xu *et al*. | The Influence of Historical Land Use and Water Availability on Grassland Restoration | 2009 | Restor. Ecol. | 18 | 217-225 |
| R.A. Distel | Restoration of palatable grasses: A study case in degraded rangelands of central Argentina | 2008 | J. Arid. Envirnon. | 72 | 1968-1972 |
| S.D. Wilson *et al*. | Semiarid old-field restoration: is neighbor control needed? | 2004 | Ecol. Appl. | 14 | 476-484 |
| W. Xing *et al*. | Grazing exclusion-induced shifts, the relative importance of environmental filtering, biotic interactions and dispersal limitation in shaping desert steppe communities, northern China | 2017 | J. Arid Land | 10 | 402-415 |
| N.A. Haby | Long-term revegetation success of severely degraded chenopod shrublands | 2017 | The Rang. Journal | 39 | 341-354 |
| S. Tabeni *et al*. | Indicators of landscape organization and functionality in semi-arid former agricultural lands under a passive restoration management over two periods of abandonment | 2016 | Ecol. Indicators | 66 | 488-496 |
| L.M. Porensky *et al*. | Arid old-field restoration: Native perennial grasses suppress weedsand erosion, but also suppress native shrubs | 2014 | Agr. Ecos. Environ. | 184 | 135-144 |
| Q. Li *et al*. | Effects of fencing on vegetation and soil restoration in a degraded alkaline grassland in northeast China | 2014 | J. Arid Land | 6 | 478-487 |
| F.A. Yannelli *et al*. | Assessing Degradation of Abandoned Farmlands for Conservation of the Monte Desert Biome in Argentina | 2014 | Environ. Manag. | 53 | 231-239 |
| K. Torok *et al*. | Long-term outcome of nitrogen immobilization to restore endemic sand grassland in Hungary | 2014 | J. App. Ecol. | 51 | 756-765 |
| M. Jankju | Role of nurse shrubs in restoration of an arid rangeland: Effects of microclimate on grass establishment | 2013 | J. Arid. Environ. | 89 | 103-109 |
| M. Tsuboa *et al*. | Plant volatiles inhibit restoration of plant species communities in dry grassland | 2012 | Basic App. Ecol. | 13 | 76-84 |
| B. Wang *et al*. | Changes in soil physico-chemical and microbiological properties during natural succession on abandoned farmland in the Loess Plateau | 2011 | Environ. Earth Sc. | 62 | 915-925 |
| S.C. Jiang *et al*. | Vegetation restoration of secondary bare saline-alkali patches in the Songnen plain, China | 2010 | App. Veg. Sc. | 13 | 47-55 |
| T. Yayneshet *et al*. | The effects of exclosures in restoring degraded semi-arid vegetation in communal grazing lands in northern Ethiopia | 2009 | J. Arid Environ. | 73 | 542-549 |
| L. Li *et al*. | Differences of arbuscular mycorrhizal fungal diversity and community between a cultivated land, an old field, and a never-cultivated field in a hot and arid of southwest China ecosystem | 2007 | Mycorrhiza |  |  |
| M.I. El-Bana *et al*. | The Importance of Phytogenic Mounds (Nebkhas) for Restoration of Arid Degraded Rangelands in Northern Sinai | 2003 | Restor. Ecol. | 11 | 317-324 |
| P.C. Beukes and R.M. Cowling | Evaluation of Restoration Techniques for the Succulent Karoo, South Africa | 2003 | Restor. Ecol. | 11 | 308-316 |
| D.R. Pérez *et al*. | Direct seeding and outplantings in drylands of Argentinean Patagonia: estimated costs, and prospects for large-scale restoration and rehabilitation | 2019 | Restor. Ecol. |  |  |
| Y. Li *et al*. | Grazing Exclusion, a Choice between Biomass Growth and Species Diversity Maintenance in Beijing-Tianjin Sand Source Control Project | 2019 | Sustainability | 11 | 1-18 |
| J. Li *et al*. | Dynamics of soil microbial C:N:P stoichiometry and its driving mechanisms following natural vegetation restoration after farmland abandonment | 2019 | Sc.Total Environ. | 693 |  |
| L. Ovsepyan *et al*. | Recovery of organic matter and microbial biomass after abandonment of degraded agricultural soils: the influence of climate | 2019 | L. Degr. Develop. |  | 1-14 |
| L. Rentao *et al*. | Conversion of cropland into agroforestry land versus naturally-restored grassland alters soil macro-faunal diversity and trophic structure in the semi-arid agro-pasture zone of northern China | 2019 | J. Arid Land | 11 | 306-317 |
| J. Liu *et al*. | Effects of different fencing regimes on community structure of degraded desert grasslands on Mu Us desert, China | 2019 | Ecol. Evol. | 9 | 3367-3377 |

**Table S2.** Spatial grain size (m^2^) for active and passive categories of restoration practices included in a meta-analysis in dryland agricultural ecosystems globally. Data are mean ± standard error.

| **Restoration practices** | **Active restoration** | **Passive restoration** |
| --- | --- | --- |
| Soil | 1008.39 ±33.62 | 431.73 ± 20.43 |
| Vegetation | 2786.17 ± 818 | 77.27 ± 13.24 |
| Water addition | 221.08 ± 7.97 | - |
| Grazing exclusion | - | 4326.78 ± 595.52 |
