# Supplementary material for: A meta-analysis contrasting active versus passive restoration practices in dryland agricultural ecosystems
Source: PeerJ. 2020 Nov 23;8:e10428. doi: 10.7717/peerj.10428 (PMC7690292; doi:10.7717/peerj.10428)
Supplement: Supplemental Information 3 [file peerj-08-10428-s003.docx]

PeerJ Article ID: 52729

Title: A meta-analysis contrasting active versus passive restoration practices in dryland agricultural ecosystems

Changes Required

1. The rationale for conducting the systematic review / meta-analysis

**Answer:**

Dryland ecosystems are an extended land cover type globally that support nearly 40% of the world’s population and are home of a variety of unique species, ecological interactions and provide important ecosystem functions. Agricultural ecosystems such as farmlands and grazing lands are also common in drylands globally. However, global water scarcity particularly agricultural dryland ecosystems impacted by overexploitation, land degradation, and climate change is increasingly driving retirement of those agricultural lands that are no longer productive. This general context provides the opportunity to re-claim some of these lands. The specific restoration goal and the availability of funding will further define the type of restoration, active or passive, applied locally or regionally.

Restoration of agricultural drylands back to habitat for plants and animals will provide capacity for reductions in species loss in these biodiversity hotspots and contribute to more secure water and food resources for a rapidly expanding human population. Therefore, the main rationale for conducting this meta-analysis is to offer knowledge to researchers and stakeholders about restoration efforts implemented in drylands formerly used to agricultural practices. Thus, we applied meta-analysis to compare the success of active and passive restoration practices globally in agricultural dryland ecosystems and to identify any general trends in the success of these practices for restoring native habitat.

2. The contribution that it makes to knowledge in light of previously published related reports, including other meta-analyses and systematic reviews

**Answer:**

The class of restoration practice to implement depends on the type and extent of damage to the ecosystem. Generally, more degraded ecosystems will require more active efforts to be restored. The focus of this meta-analysis is to evaluate restoration of agricultural dryland ecosystems globally.

Previous meta-analyses in tropical and temperate rain forests shown that passive restoration including natural succession processes can lead to positive, desired plant and animal restoration outcomes. However, for drylands, this meta-analysis showed that active restoration practices more consistently led to positive restoration outcomes. This can be explained by the physical constraints of dryland ecosystems that experience relatively severe limitations in rainfall, soil fertility, and productivity. Therefore, this evidence highlights the need for consideration of environmental limitations in drylands for conducting restorative projects.

Results of this meta-analysis showed that active restoration practices are mainly required to achieve soil-based outcomes in agricultural drylands, while passive practices lead to negative soil restoration outcomes. This is consistent with previous work from the San Joaquin Desert of California that recommends any restoration project on formerly farmed lands start with soil nutrient remediation. Thus, active restoration may be necessary to overcome the legacies of soil disturbances, nutrient additions, and pesticide usage in agricultural drylands. The extent of land transformation and the type of prior land use can also contribute to the requirement of increased efforts and investments to achieve agricultural dryland restoration.
